# A Long Time-Series Radiometric Normalization Method for Landsat Images

**DOI:** 10.3390/s18124505

**Published:** 2018-12-19

**Authors:** Wei Wu, Xia Sun, Xianwei Wang, Jing Fan, Jiancheng Luo, Ying Shen, Yingpin Yang

**Affiliations:** 1 College of Computer Science and Technology, Zhejiang University of Technology, Hangzhou 310023, China; wuwei@zjut.edu.cn (W.W.); sunxia528@163.com (X.S.); fanjing@zjut.edu.cn (J.F.); shenying@zjut.edu.cn (Y.S.); 2 Center for Global Sea Level Change, New York University Abu Dhabi, Abu Dhabi 129188, UAE; xw21@nyu.edu; 3 Institute of Remote Sensing and Digital Earth, Chinese Academy of Sciences, Beijing 100101, China; yangyp@radi.ac.cn; 4 University of Chinese Academy of Sciences, Beijing 100049, China

**Keywords:** radiometric normalization, long time-series, cloud and cloud shadow, pseudo-invariant features, inflexion-based cloud detection

## Abstract

Radiometric normalization attempts to normalize the radiomimetic distortion caused by non-land surface-related factors, for example, different atmospheric conditions at image acquisition time and sensor factors, and to improve the radiometric consistency between remote sensing images. Using a remote sensing image and a reference image as a pair is a traditional method of performing radiometric normalization. However, when applied to the radiometric normalization of long time-series of images, this method has two deficiencies: first, different pseudo-invariant features (PIFs)—radiometric characteristics of which do not change with time—are extracted in different pairs of images; and second, when processing an image based on a reference, we can minimize the residual between them, but the residual between temporally adjacent images may induce steep increases and decreases, which may conceal the information contained in the time-series indicators, such as vegetative index. To overcome these two problems, we propose an optimization strategy for radiometric normalization of long time-series of remote sensing images. First, the time-series gray-scale values for a pixel in the near-infrared band are sorted in ascending order and segmented into different parts. Second, the outliers and inliers of the time-series observation are determined using a modified Inflexion Based Cloud Detection (IBCD) method. Third, the variation amplitudes of the PIFs are smaller than for vegetation but larger than for water, and accordingly the PIFs are identified. Last, a novel optimization strategy aimed at minimizing the correction residual between the image to be processed and the images processed previously is adopted to determine the radiometric normalization sequence. Time-series images from the Thematic Mapper onboard Landsat 5 for Hangzhou City are selected for the experiments, and the results suggest that our method can effectively eliminate the radiometric distortion and preserve the variation of vegetation in the time-series of images. Smoother time-series profiles of gray-scale values and uniform root mean square error distributions can be obtained compared with those of the traditional method, which indicates that our method can obtain better radiometric consistency and normalization performance.

## 1. Introduction

Remote sensing satellites observe the land surface of the Earth at regular time intervals with the same observation geometry and obtain time-series of images, which record the occurrence and development patterns of land surface phenomena and thus have been widely applied for land change detection [1], crop yield estimation [2], urban sprawl analyses [3], land-cover transition evaluations [4], and forest succession analyses [5], and achieving great success. Despite the great success of applications based on time-series of images, the physical signal recorded by a remote sensor (such as gray-scale value or reflectance) at different dates is inevitably contaminated by noise unrelated to the land surface, including different atmospheric conditions at the time of image acquisition and sensor distortion, which can cause variations in radiometric features between images and decrease the comparability between different images over the same study area [6]. Contaminated signals can lead to sharp increases or decreases in the profiles of time-series indicators such as vegetation indices, which conceals the actual changes of the land surface and hinders information extraction. Thus, removing radiometric distortion is urgently needed to facilitate remote sensing applications.

Radiometric calibration attempts to eliminate the radiometric distortion caused by non-surface factors and correct radiometric differences between different images. Based on the transformation of gray-scale values to physical signals, calibrations can be classified into absolute radiometric calibrations and relative radiometric calibrations [1]. An absolute radiometric calibration establishes the relationship between the measurement values from a remote sensor and the reflectance of the land surface to eliminate the radiometric distortion between images. This method needs to establish an “atmosphere–land surface–sensor” interaction model, involving certain environmental parameters (such as the atmosphere) at acquisition time [7]. Additionally, some pseudo invariant calibration sites are required to calibrate the on-orbit sensors [8]. However, not all archived historical data were recorded with environmental information, which restricts the practicability of this method [5,9]. The relative radiometric calibration uses a certain image as a reference and corrects another image based on the reference; thus, the processed image will have a similar radiometric condition as the reference for the same land surface, namely, radiometric normalization. Radiometric normalization directly establishes the mapping relationship of radiometric features between different images and can obtain an application effect comparable to that of absolute radiometric calibration [5,10]. Currently, there are primarily two types of methods for radiometric normalization; mapping and regression methods.

The mapping method directly establishes a gray-scale mapping equation between images and uses the mapping value to replace the gray-scale value of the input image. For instance, using a linear equation, the mean and standard deviation of a reference image can be assigned to the image to be processed, and the processed image therefore has the same average and standard deviation as the reference image, which eliminates the radiometric distortion. Another widely used mapping method is histogram specification, which assigns the histogram of the reference image to the input image so that the processed image has the same gray-scale distribution as the reference image [11]. Because different bands of a multispectral image are usually correlated, the differences in the radiometric features can be eliminated by defining a high-dimensional rotation matrix to match the density function in multidimensional space [12]. Radiometric normalization based on histogram specifications has been used for land surface change detection [13], gap filling [14], and image mosaicking [11].

The regression method establishes a regression model to describe the radiometric distortion relationship between the images through pseudo-invariant features (PIFs), which represent pixels with radiometric features that do not change with time, such as those of buildings and bare land [15]. As the radiometric difference between PIFs are mainly attributed to noise factors, we can establish a regression model to quantify the radiometric difference between different images and eliminates the radiometric difference according to the obtained regression model. As a result, the selection of high quality PIFs is the key for radiometric normalization. The PIF selection method mainly includes principal component analysis [16], weighted principal component analysis [17], multivariate alteration detection (MAD) [18], improved Iteratively Re-Weighted MAD (IRMAD) [9], and iterative slow feature analysis [19]. The PIF extraction methods based on categories, for example, the temporally invariant cluster (TIC) method [1], are more robust than that of pixel-level methods. For radiometric normalization models, linear regression models are simple and effective, and later improved linear regression methods such as the ordinary least-squares regression, reduced major axis regression [20] and Theil-Sen regression [21] have been successively developed and widely used to correct radiometric distortions. In addition, artificial intelligence methods, for example, genetic algorithms, can be used to not only optimize the regression parameters but also eliminate nonlinear distortions [22].

According to the comparison of the two methods above, the mapping method changes variations in the radiometric features caused by atmospheric and sensor factors as well as land surface information. The physical meaning of the corrected image is not definitive; therefore, the mapping method is mainly suitable for generating visually seamless image mosaics [11] or change detection [13]. However, the regression method selects PIFs that are affected only by atmospheric and sensor factors and establishes a model to describe land surface radiometric variation [15]. The regression method can maintain land surface information and eliminate image-related radiometric distortion, and these merits make it an ideal method for radiometric normalization of long time-series of images for land surface applications.

However, traditional PIF extraction and regression equation establishment methods are developed for two images and operate in an image-to-image manner [1,9,15]. Although virtual reference image determination methods [23] and overall adjustments [16] have been developed, when these methods are extended to radiometric normalization for long time-series of images, they also operate in an image-to-image manner [10]. For a radiometric normalization problem involving time-series of images with *n* scenes, we should subdivide it into a group of *n* − 1 image-to-image subproblems. Obviously, these extensions have two deficiencies. (1) The PIFs are extracted for each group of images separately, which may not be consistent between different groups of images; therefore, the correction models among different groups may not be comparable. Additionally, the invariance of different surface features has different time scales. The traditional selection of PIFs considers the changes of radiometric features at only two times and causes a potential error in PIF selection. (2) When an image is processed based on a reference image, although the radiometric residual in between can be minimized, but the residual for the correction between any two scenes of the images to be processed cannot be minimized. The time-series analysis views all images in the time-series as a continuously varying entity, thereby minimizing the radiometric residual for the overall correction between any two image scenes represents an optimal solution.

In recent years, with the development of computing technology and the free distribution of medium resolution images through the internet, applications utilizing long time-series of images can provide more temporal details with high accuracy and satisfy various demands; thus, they have become a popular research topic in remote sensing fields [24,25,26]. However, according to the above analysis, traditional radiometric normalization is unsuitable for long time-series of images, and, therefore, a radiometric normalization method suitable for long time-series of images must be developed. To solve this problem and meet application demands, we will try to identify a better method for radiometric normalization of long time-series of remote sensing images in this paper. The main contribution of our method is twofold:

- we developed a PIF selection method, which can consider all images in time-series for PIF selection and automatically suppress the negative effective of outliers, for example, clouds and cloud shadows; and
- a novel optimization strategy is proposed to minimize the residual between the image to be processed and the images that have been processed previously, which can avoid the problem of reference image selection and obtain a smoother time-series profile.

The remaining part of the paper is structured as follows: Section 2 introduces the research area and the experimental data; Section 3 describes the principle of our method; Section 4 introduces the implementation approach in detail; Section 5 introduces the experimental results and their performance comparison; Section 6 discusses the applicability of our method and its uncertainty; and Section 7 presents the conclusions.

## 2. Materials

**Figure 1:**
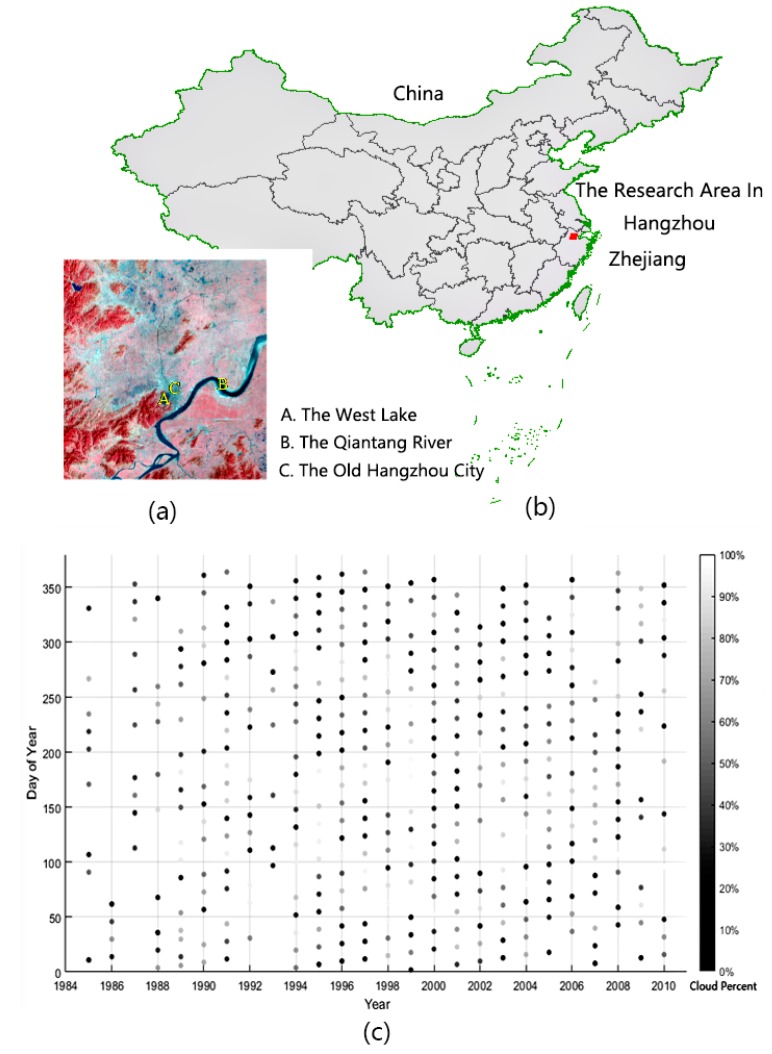
(**a**) Image acquired on March 3, 1986, of the research area, Hangzhou City. (**b**) Location of Hangzhou City (red square) in Zhejiang Province, China. The border of China is indicated with green polygons, and the boundaries of different provinces are indicated with gray polygons. (**c**) Image acquisition dates and proportions of noise caused by clouds and cloud shadows. All the images were taken by Landsat 5.

Hangzhou City in Zhejiang Province, China, is selected as our study area because of its rapid land use and cover changes and intensive urbanization in the last thirty years [27]. The research area is indicated in Figure 1b. To demonstrate our radiometric normalization method, we selected Thematic Mapper (TM) images from Landsat 5 with Path = 119 and Row = 38 in the Worldwide Reference System (WRS) for the experiment. From the website of the United States Geological Survey (USGS), we obtained a total of 438 scenes of image covering Hangzhou City from 1984 to 2010, the entire operational period of Landsat 5 (the distribution of the imaging dates is shown in Figure 1c). Then, we conducted preprocessing that included image decompression and band synthesis, and a subregion of 2000 × 2000 (pixels) was clipped for our experiment. No further geometric processing was conducted because the geolocation accuracy of these images from the USGS was better than one pixel. The goal of this paper is to find a better method of radiometric normalization; therefore, we directly adopted the gray-scale values of different bands to process without converting them to physical signals such as reflectance. The gray-scale depths of the points in Figure 1c represent the coverage proportion of noise (such as clouds) in each image (the detailed mask method will be described in the Section 4.2). We can see that the proportion of clouds is approximately 50% on average, and the identification of cloud noise in the image is thus the basis for subsequent analyses of the time-series of remote sensing images.

**Figure 2:**
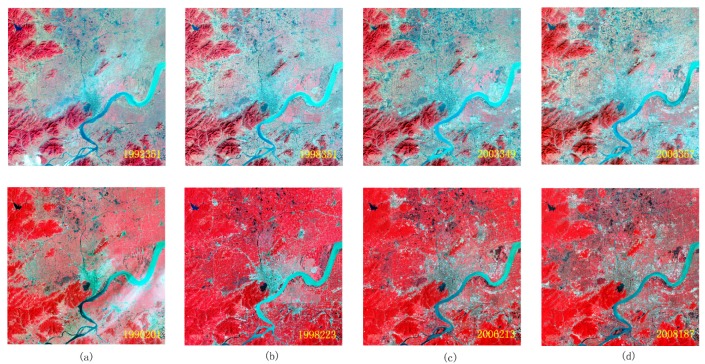
Time-series of remote sensing images from Landsat 5 over Hangzhou City. (**a**)–(**d**) represents four image acquired on 1990, 1998, 2003, and 2008, respectively, the images from the upper row were obtained on approximately the 350th days of different years, and those of the lower row were obtained on approximately the 205th days of different years.

After looking through all the images, the typical false-color composite images over Hangzhou City during four different time periods (1990, 1998, 2003, and 2008) are shown in Figure 2. The built-up area of Hangzhou City in 1990 was mainly concentrated in the periphery of West Lake, and some villages and towns were distributed discretely in the research area. However, the land cover type in the suburb of Hangzhou City was mainly agricultural land. In approximately 2000, the large amount of farmland around Hangzhou City was transformed into cultivated land, and there was only a small amount of farmland remaining around Hangzhou City. In 2010, the extent of the built-up district expanded. Except for the mountains in the west of the research area, where it was difficult to use the land for construction, almost all the land was converted to urban buildings [27]. The land use/cover changes of Hangzhou City indicates that the selection of PIFs is difficult for time-series of remote sensing images because the number of PIFs continues to decrease during long time periods. A better method of finding PIFs over long time-series of remote sensing images will benefit radiometric normalization and land use/cover analysis.

## 3. Principles

### 3.1. Factors That Induce Radiometric Variation in Time-Series of Images

We first create a data set for all the images over Hangzhou City from Landsat 5. The *n* scenes of images obtained at different times in a certain research area are sorted by acquisition date to constitute the time-series of the image set *X*:

(1) $$X=\{X_{1},X_{2},...,X_{n}\}.$$

The set of pixels corresponding to the image region can be expressed as *L*, and the time-series of observation values $P^{l}$ for a pixel (l∈L) is

(2) $$P^{l}=\{P_{1}^{l},P_{2}^{l},...,P_{n}^{l}\},$$

where $P_{i}^{l}$ represents the gray-scale value of pixel *l* in the image $X_{i}\in X$, and $P^{l}$ describes the temporal evolution of the corresponding land surface region, which can be decomposed into three main components: trend, seasonal change, and remaining components [28]. Similar to Yuan [29], the factors that influence radiometric characteristics of long time-series of pixels with $P^{l}$ over the same area can be summarized into five types.

(1) Image acquisition condition: Atmospheric conditions and solar elevation at the time of image acquisition will pose a direct influence on the radiometric characteristics of the acquired image.
(2) Sensor distortion: The performance of a sensor will decay over its operational lifetime, causing the measurement values obtained by the sensor to decrease over time. However, within a certain time period, the radiometric properties of the sensor, especially the TM Sensor onboard Landsat 5 [30], can be considered relatively stable.
(3) Abnormal observation: Clouds and cloud shadows result in extremely large and small values on images, respectively, and the variation magnitude of the corresponding gray-scale values is much larger than that of clear-sky pixels. In observations at adjacent times, the gray-scale value of abnormal observations over the same land surface is prone to change steeply.
(4) Seasonal change of vegetation: This process is affected by the physiological processes of vegetation growth and withering, which exhibit an annual cyclic variation. Additionally, there are interannual time changes of delays or leading, and magnitude changes of intensifying or weakening in the observation measurements.
(5) Land cover change: This change is induced by the land cover changes, including changes in land use or natural conditions.

From the above analysis, type (1) and (2) factors form the main noise source of remote sensing images, and this noise conceals variations of the land surface and should be eliminated through radiometric normalization. The clear-sky PIFs (i.e., without any cloudy or noisy effect) are affected by these factors only, so we can model the effect of these factors with a linear equation using the clear-sky PIFs. Assuming that one image in the time-series is similarly affected by these factors, we can eliminate the effect using the obtained linear equation. Therefore, the problem of radiometric normalization has been transformed to the selection of clear-sky PIFs based on abnormal observations (i.e., factor of type (3)), seasonal evolution of vegetation (i.e., factor of type (4)), and land cover change (i.e., factor of type (5)) exclusion.

### 3.2. Variation Range of Different Land Surfaces

As observed by Liu [31], the gray-scale values of the same pixel are similar during the same season of different years or in adjacent days under similar climate conditions. However, the gray-scale values of clouds usually vary greatly at the same location, even in adjacent days. In short, the variation of gray-scale values for clear pixels is usually low and changes slowly from day to day compared with that for clouds, which is generally high and varies over a wide range. If the long time-series’ gray-scale values assembled from the same location are sorted in ascending order, the clear-sky observations in that queue will be located at the front portion, and the cloudy observations will be collected in the back portion. As the gray-scale values of clear-sky observations distribute within a narrow range, cloudy pixels distribute over a wide range, the slope of the clear-sky portion in the sequence is small, and the slope of the cloudy portion in the sequence is large. An inflexion point exists at the transition from the clear-sky observations to the cloudy observations. According to this fact, an inflexion-based cloud detection (IBCD) algorithm has been developed for generating cloud masks for Moderate Resolution Imaging Spectroradiometer (MODIS) land surface reflectance products [31].

**Figure 3:**
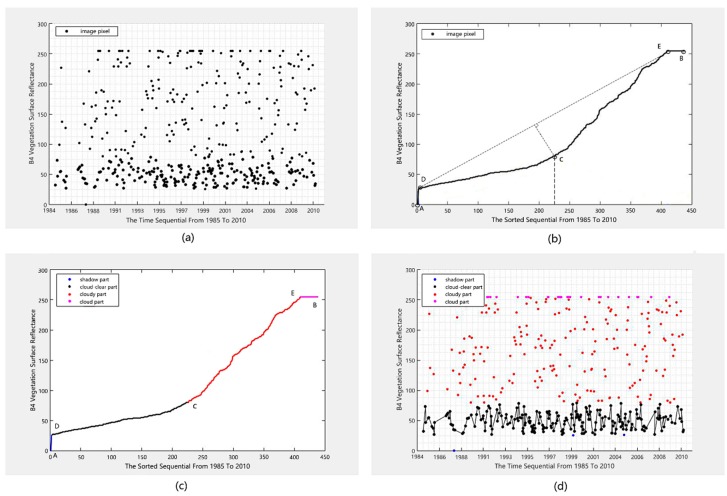
Schematic diagram of the time-series pseudo-invariant feature (PIF) extraction method based on the point of inflection. (**a**) Original gray-scale values for a typical selected pixel sorting according to their acquisition times; (**b**) gray-scale values sorted in ascending order, with A and B representing points with the smallest and largest gray-scale values, respectively. C, D and E are identified inflection points. (**c**) Segmentation results of a line segment using the sorted gray-scale values; and (**d**) inlier and outlier identification results using the method proposed in this paper.

**Figure 4:**
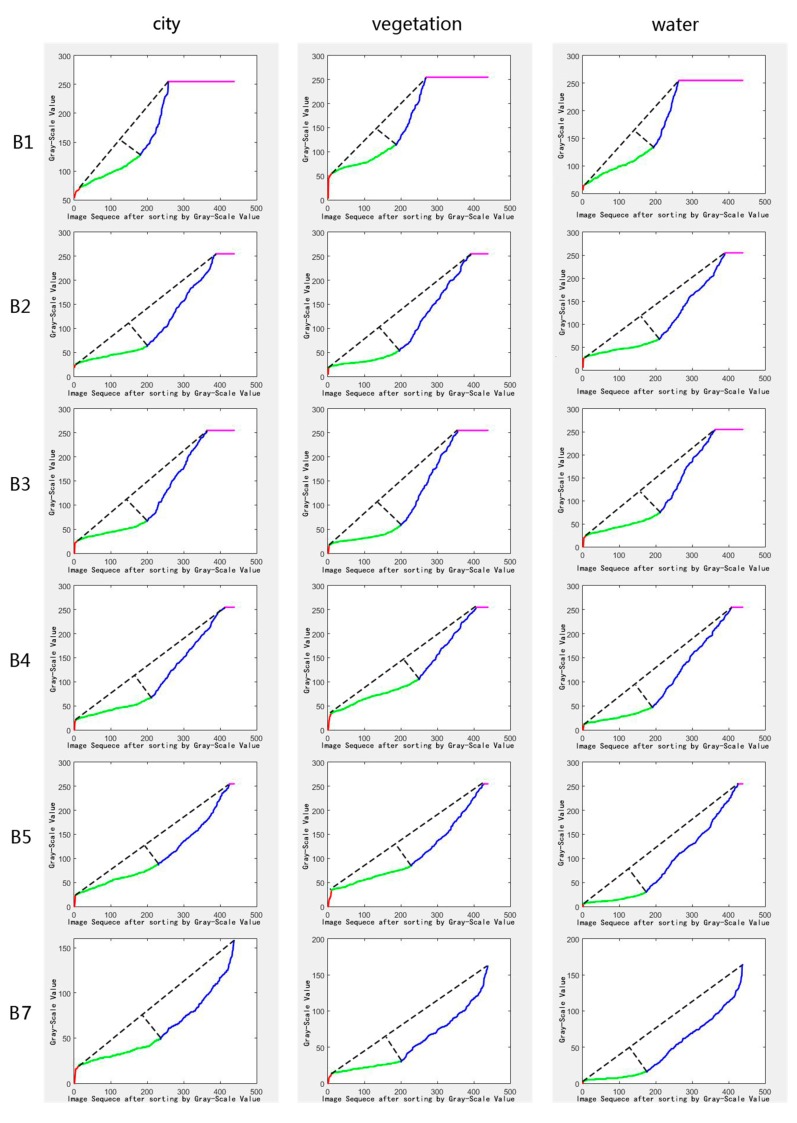
Schematic diagram for the variations of the time-series’ gray-scale value curves in multiple bands for three different land surface types (city, vegetation, and water body). The normal observation (indicated by green line) and abnormal observation values (indicated by red, blue and pink line) have obvious points of inflection at the visible (**B1–B3**) and near-infrared bands (**B4**), however this feature is not obvious for the two short-wave infrared bands (**B5** and **B7**). The dashed lines connect the smallest gray-scale values, clear pixels and largest cloudy pixels, and the inflection points can be determined by searching the maximum distances from the arcs to the dashed lines.

Figure 3a shows the original gray-scale values of the near-infrared (NIR) band for a typical selected pixel in our study area, sorted according to their acquisition times. Figure 3b illustrates gray-scale values sorted in ascending order, and we denote the curve composited by these points as Arc. Additionally, we can observe five feature points in the Arc (including the start point *A*, end point *B*, and three inflection points indicated by *C*, *D*, and *E*), which split the Arc into four segments as illustrated in Figure 3c. Three typical pixels (city, vegetation, and water), sorted in ascending order according to their gray-scale values, are shown in Figure 4 with the six bands of TM (exclusion of thermal infrared i.e., B6), and the five feature points have clipped the Arc into four segments indicated by different colors. According to reference [31], the red or blue line segment indicates observations under cloud influence, which has the largest variation magnitude and exhibits a steep increase in the gray-scale value curve time-series. However, for the normal observation values (namely, clear pixels) of the green line segment, the variation range is small, and the change of the time-series of the gray-scale value curve is slow. A distinctive inflexion point occurs at the transition from the clear-sky observations to the cloudy observations. Accordingly, we can separate the clear observations from the cloudy observations using a modified IBCD method.

After excluding the abnormal values caused by clouds and cloud shadows from the time-series observations, the main challenge of long time-series of radiometric normalization has transformed to PIF selections. The magnitude of gray-scale value variations in the time-series of observations of a pixel caused by different factors is not consistent, for example, radiometric variation caused by land surface change, which is larger than that of other factors, and these variations form the theoretical basis for threshold-based change detection [32]. The time-series of PIF variations can be considered as a stationary time-series, the unconditional joint probability distribution of which does not change over time. In other words, parameters such as the mean and variance also do not change over time [33]. In an ideal situation, the time-series values of pixels would obey the normal distribution, and most of the gray-scale values would be concentrated around the mean, which has been used for PIF selection [9,15,18]. In contrast, vegetative pixels are influenced by intra-year seasonal changes and inter-year amplitude changes, and the time-series’ gray-scale values vary within a wide range. This fact has been verified by classical and recent studies of time-series decompositions [30].

Specifically, the variation magnitude of PIFs is smaller than that of vegetation and land cover change pixels. Figure 4 also shows that on the line segment composed of normal observation values (the green line segment, which is called the clear line segment hereafter). In the visible light (B1, B2, and B3) and near-infrared bands (B4), the variation amplitudes of water bodies, cities (namely, PIFs), and vegetation continue to increase, and the line segments change from gentle to steep (i.e., the slopes of the line segments change from small to large), however this feature is not obvious for the two short-wave infrared bands (B5 and B7). This feature is particularly obvious in the near-infrared band (B4, in Figure 4). Therefore, we can identify the PIFs based on the time-series’ gray-scale value curve segmentation. In summary, the clear line segment of the PIFs is steeper than that of water bodies and flatter than that of vegetation and land surface change pixels. This characteristic can be quantitatively expressed with the slope of the clear line segment; thus, the slope of PIFs is smaller than that of vegetation and larger than that of water bodies.

## 4. Methods

**Figure 5:**
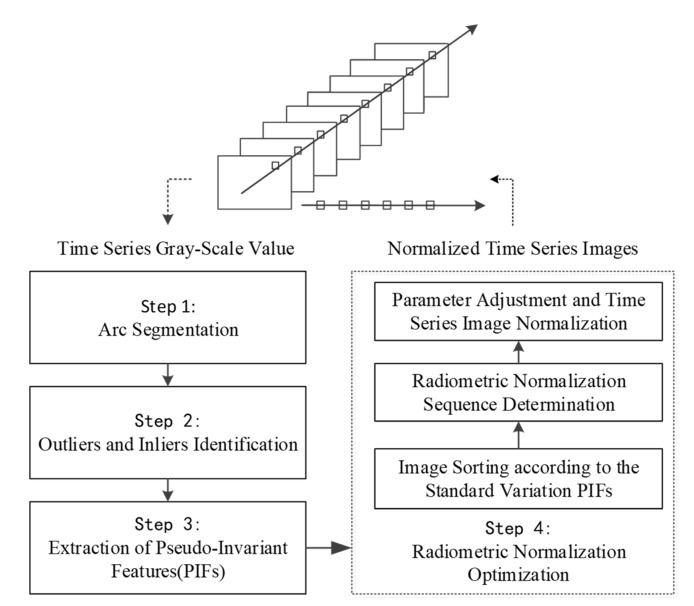
Flowchart of a long time-series radiometric normalization method for Landsat images proposed in this paper.

According to the analysis above, after segmenting the Arc into segments, our method can be implemented using the following steps (as Figure 5). First, we classified the time-series’ observation values for every pixel into inliers (namely, normal observation values with good acquisition condition) and outliers (namely, abnormal observation values caused by cloud and cloud shadow noise). Second, PIFs were extracted based on the smaller and larger variation magnitudes of PIFs relative to those of vegetation and water, respectively. Next, we established the radiometric calibration equation for images acquired at different time points to eliminate the radiometric distortion between images. Therefore, we will introduce our method with four main steps as follows.

### 4.1. Arc Segmentation

For the time-series of observations *P^l^* for pixel *l* (l∈L) (as shown in Figure 3a), we first segmented the Arc into segments using the following method.

Step 1.1: Sorting the time-series’ gray-scale values: The time-series’ gray-scale values of *P^l^* for pixel l∈L were sorted in ascending order to generate a series of sequence values, which is expressed as $P_{S}^{l}$:

(3) $$P_{S}^{l}=<P_{S1}^{l},\;P_{S2}^{l},\;...,\;P_{sn}^{l}>.$$

The sorting results are shown in Figure 3b with the black solid line. This arc is denoted as $\mathrm{Arc}(P^{l})$, and the points in $\mathrm{Arc}(P^{l})$ can be expressed as

(4) $$V=<v_{1},\;v_{2},\;...,\;v_{n}>.$$

The coordinates of points $v_{i}\in V$ on $\mathrm{Arc}(P^{l})$ can be expressed as $(x_{s_{i}},y_{s_{i}}),i=1,\;2,\;...,\;n$. Here, $x_{s_{i}}$ represents the image sequence after the observation values are sorted and is a natural number in the range of [1, *n*]; and $y_{s_{i}}$ represents the gray-scale value corresponding to $x_{s_{i}}$, which ranges from 0 to 255 for the TM image.

Step 1.2: Arc Segmentation with the inflection points: Connect point $v_{1}$, which is marked by *A* in Figure 3b and has a minimum gray-scale value, and $v_{n}$, which is marked by *B* in Figure 3b and has a maximum gray-scale value, to determine the straight line $l(P^{l})$. The equation of the straight line can be expressed as

(5) ax+by+c=0.

According to Equation (6), we calculate the distance $d(v_{i})$ for point $v_{i}\in V$ in the arc $\mathrm{Arc}(P^{l})$ to the straight line $l(P^{l})$:

(6) $$d(v_{i})=\frac{\left|ax_{si}+by_{si}+c\right|}{\sqrt{a^{2}+b^{2}}}.$$

The distances of all the points in the $\mathrm{Arc}(P^{l})$ form a vector:

(7) $$DIS=\{d(v_{1}),\;d(v_{2}),\;...,\;d(v_{n})\}.$$

Obviously, the point of inflection on $\mathrm{Arc}(P^{l})$ corresponds to the maximum value in the distance vector. Accordingly, the inflection point *C* can be obtained by extracting the maximum distance in

(8) $$v_{c}=\underset{v_{i}\in V}{\arg\max}d(v_{i}).$$

Connect points *A* and *C*, and then points *B* and *C*, and then repeat the step mentioned above. Then, we can obtain two other inflection points named *D* and *E*. According to the inflection points obtained, we can split $\mathrm{Arc}(P^{l})$ into four segments as shown in Figure 3c, which can be expressed as, *AD*, *DC*, *CE*, and *EB*.

### 4.2. Outliers and Inlier Identification

Step 2.1: Marking outliers and inliers: We took the segment comprising the minimum values (namely, the blue segment in Figure 3c) as the abnormal observation value affected by a cloud shadow. The segment in the middle part (namely, the black segment in Figure 3c) represents the normal observation value, and we denoted it as a clear line segment. The two segments of and with maximum gray values (namely, the red and pink segment in Figure 3c) are marked as the abnormal observation values affected by cloud.

### 4.3. Extraction of Pseudo-Invariant Features 

Step 3.1: Slope calculation for the clear line segment: First, we conducted a least squares fitting on the clear line segment and then used the slope to express the inclination degree of various line segments. Then, the research area can be expressed with one band of *B_S_* indicating the slope of the clear line segment of the pixel, which describes the gray-scale value variation magnitude.

Step 3.2: Extraction of PIFs: We set two thresholds *T_Low_* and *T_Hig_* and took the values of *B_S_* greater than *T_Low_* and smaller than *T_Hig_* as the PIFs. Here, the selection of the thresholds was not obtained by a universal criterion or an automated method. Instead, we selected the threshold manually to ensure the representativeness and integrity of the PIFs by statistically analyzing and observing the characteristics of *B_S_*, adjusting *T_Low_* and *T_Hig_*, and using the trial-and-error method. 

More specifically, by analyzing the histogram of the Band *B_S_* (transformed to bins of integers), we defined two thresholds to exclude the land surfaces with low slopes (such as water) and land surfaces with high slopes (such as vegetation and changed land cover) first. We then segmented the remaining pixels into certain predefined groups. Then, through investigating the pixels in the various groups, we selected minimum and maximum gray-scale values of the group of the most invariant pixels (PIFs candidates) as the initial *T_Low_* and *T_Hig_* , and then adjusted the *T_Low_* and *T_Hig_* to exclude or include some pixels as PIFs.

Step 3.3: Exclusion of cloudy images with small amounts of clear PIFs: It is worth noting that images containing high proportions of clouds and cloud shadows can bring in noisy pixels. When using these cloudy images, the numbers of PIFs from different images substantially differ, and it is difficult to obtain sufficient PIFs to estimate a reliable radiometric normalization equation. In this study, we excluded these cloudy images by using two conditions, minimum clear PIFs number (CPmin) and the coefficient of determination ($R^{2}$) between the image to be processed and the first image (the method for determination of the first image will be introduced in Step 4.2). If the $R^{2}$ was smaller than the threshold, the corresponding images were excluded from further radiometric normalization.

According to this method, we could obtain PIFs with relatively small changes in land surface features during the entire image acquisition period, and the obtained *m* PIFs are expressed as a set *S*. Obviously, *S* is a subset of *L*, namely, S⊆L.

### 4.4. Radiometric Normalization Optimization

When the least squares method is adopted to normalize the image $X_{i}\in X$ based on the reference image $X_{R}\in X$, the objective is to obtain normalized images denoted by $f(X_{i})$, which minimizes the correction error between $f(X_{i})$ and $X_{R}$. This process is equivalent to solving the optimal solution under the constraint of the objective function $Q_{R}$:

(9) $$Q_{R}=\min\sum_{i=1}^{n}\sum_{s\in S}{\left\|f(X_{i}^{s})-X_{R}^{s}\right\|}_{2},$$

where $f(X_{i})$ represents the radiometric normalization result of the image $X_{i}$, ${\left\|•\right\|}_{2}$ represents the 2-norm of the vector, and s∈S represents the selected PIFs. Clearly, this model cannot ensure an optimized solution with the smallest residual for all the corrected images.

Hence, in this paper, we propose a time-series normalization strategy under the constraint of the objective function $Q_{G}$:

(10) $$Q_{G}=\min\sum_{i=1}^{n}\sum_{j=1}^{n}\sum_{s\in S}{\left\|f(X_{i}^{s})-f(X_{j}^{s})\right\|}_{2}.$$

This objective optimization model not only minimizes the error between the normalized image and the reference image but also minimizes the error between the image to be processed and the image these were processed previously. As a result, a radiometric consistent time-series of images will be obtained. To find the optimal solution for the objective function $Q_{G}$, we designed the following algorithm.

Step 4.1: Sorting the standard deviations for PIFs: We calculated the standard deviation of the gray-scale value for the sample set *S* for the *n* scenes of time-series images and sorted them in descending order. The standard deviation of the gray-scale value after sorting can be expressed as

(11) $$\sigma_{r}=\{\sigma (r_{1}),\;\sigma (r_{2}),\;...,\;\sigma (r_{n})\}.$$

Obviously, the standard deviation satisfies $\sigma_{ri}\geq \sigma_{r(i+1)},i=1,2,...,n-1$. The sequence numbers of the images are sorted according to standard deviation in descending order, which can be expressed as

(12) $$r=\{r_{1},\;r_{2},\;...,\;r_{n}\}.$$

Step 4.2: Correction of the first image: We first corrected image $r_{2}$ to image $r_{1}$. For implementation, we obtained the correction parameters of $k_{2}$ and $b_{2}$ under the restricted condition $Q(r_{2})$.

(13) $$Q(r_{2})=\min\sum_{s\in S}{\left(f(x{\left(r_{1}\right)}^{s})-f(x{\left(r_{2}\right)}^{s})\right)}^{2}=\min\sum_{s\in S}((k_{1}x{\left(r_{1}\right)}^{s}+b_{1})-(k_{2}x{\left(r_{2}\right)}^{s}+b_{2}){)}^{2}.$$

The restricted condition minimizes the error between corrected image of image $f(X(r_{2}))$ and the corrected image $f(X(r_{1}))$ of image $r_{1}$; this problem can be solved using the least squares method. Note that we denoted the image $r_{1}$ as $f(X(r_{1}))$ for descriptive simplicity, the correction parameters of which are $k_{1}=1$ and $b_{1}=0$.

Because the standard deviation for the gray-scale values of $r_{1}$ and $r_{2}$ satisfies $\sigma (r_{1})\geq \sigma (r_{2})$, the correction coefficient of $k_{2}$ is expected to be greater than or equal to 1 in most situations; namely, the gray-scale value of the correction results is relatively stretched. Therefore, the compression of the gray-scale value was avoided in the correction process.

Step 4.3: Correction of other images in the time series: For image $X(r_{i}),i>2$ in the time-series, we set the reference image $X{\left(r_{i}\right)}^{ref}$ for $X(r_{i})$ as follows:

(14) $$X{\left(r_{i}\right)}^{ref}=\{f(X(r_{1})),f(X(r_{2})),f(X(r_{3})),...,f(X(r_{i-1})\}.$$

The implementation method takes all the corrected images as reference images. The correction parameters of $k_{i}$ and $b_{i}$ for image $X_{r_{i}}$ can be obtained under the restricted condition:

(15) $$Q(r_{i})=\min\sum_{j=1}^{i-1}\sum_{s\in S}{\left(f(x{\left(r_{k}\right)}^{s})-f(x{\left(r_{i}\right)}^{s})\right)}^{2}=\min\sum_{j=1}^{i-1}\sum_{s\in S}{\left((k_{j}x{\left(r_{k}\right)}^{s}+b_{j})-(k_{i}x{\left(r_{i}\right)}^{s}+b_{i})\right)}^{2}.$$

Obviously, this implementation method ensures the minimum error between the image to be processed and all the corrected images and is thus a greedy algorithm. Accordingly, the correction coefficient of image $X(r_{i})$ can be obtained using the least squares method.

We repeated this step for all the images in the time-series with the order determined by (12), and we obtained the correction coefficients for various images. The correction vector can be expressed as follows:

(16) $$K={\left(k_{1},\;k_{2},\;...,\;k_{n}\right)}^{T},$$

(17) $$B={\left(b_{1},\;b_{2},\;...,\;b_{n}\right)}^{T}.$$

Step 4.4: Adjustment of correction parameters: As mentioned above, in the linear model, when the slope is $k_{i}<1,i=1,2,...n$, the radiation resolution will be compressed. When $b_{i}<0,i=1,2,...,n$, the gray value will be negative [16]. Assume that $k_{\min}$ is the minimum of

(18) $$k_{\min}=\underset{i=1,..,n}{\min}k_{i}.$$

From this, we can calculate

(19) $$b_{\min}=\frac{1}{k_{\min}}\underset{i=1,..,n}{\min}b_{i}.$$

If $k_{\min}$ < 1, we adjust *K* according to the following method and derive the new slope vector $K_{N}$:

(20) $$K_{N}=\frac{1}{k_{\min}}{\left(k_{1},\;k_{2},\;...,\;k_{n}\right)}^{T}.$$

If $b_{\min}$ < 0, we adjust *B* and obtain the new intercept vector $B_{N}$:

(21) $$B_{N}=\frac{1}{k_{\min}}{\left(b_{1}-b_{\min},\;b_{2}-b_{\min},\;...,b_{n}-b_{\min}\right)}^{T}.$$

It is worth noting that our method performs sorting according to the standard deviation to ensure that the slope value is greater than 1, and a slope less than 1 is therefore less likely to appear. Additionally, the parameter adjustment will have a negative effect on calculating the error (the details will be analyzed in Section 5.2.2). Therefore, we could omit this step decided by the user according to that $k_{\min}$ was near 1 or.

According to the correction coefficients $K_{N}$ and $B_{N}$ after adjustment, the near-infrared band of the image was corrected, and the resultant image of the radiometric normalization could obtain a smaller residual. According to the PIFs marked by this process and the corrected order, we then normalized the other bands of the image to obtain the resultant image from radiometric normalization.

## 5. Results

### 5.1. Experimental Results

**Figure 6:**
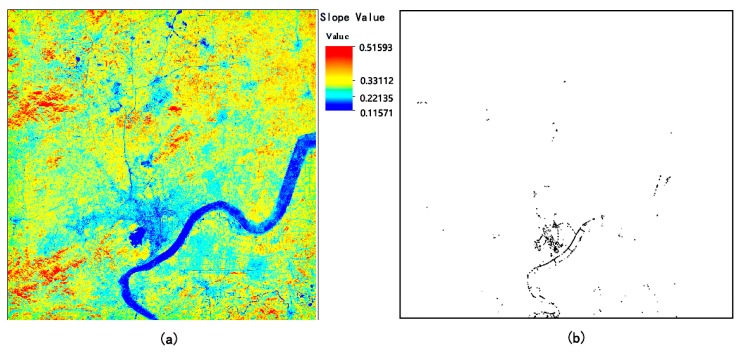
(**a**) Slope of the clear line segment, i.e., the band of *B_S_*. (**b**) PIFs extracted using the threshold segmentation from the slope of the clear line segment. The PIFs are dilated with two pixels. The black pixels indicate PIFs, and the white pixels indicate non PIFs; the selected PIFs are mainly the old Hangzhou City and artificial river levee, which remained stable during the image period.

The slope of the clear line segment *B_S_* in the research area, which forms the basis for PIF selection, is shown in Figure 6a, which shows that: (1) Water bodies presented the smallest variations and are represented by a dark blue color in the images. (2) This was followed by the PIFs of cities. The old town area of Hangzhou City and the built-up area of the surrounding villages and towns remained stable during the entire time-series and exhibit a light blue color. These areas provide redundant PIF candidates. (3) Vegetation surface features presented the largest variations. On the right side of the research area (Figure 6a), some farmland had transformed into a built-up area, and the slope of which lies between the PIFs and vegetation, whose corresponding area exhibits a bright yellow color.

The thresholds utilized in this paper for segmenting the band of slope made up of clear sky points (*B_S_*) to extract PIFs were 0.195 for *T_Low_* and 0.217 for *T_Hig_*. The PIF selection results are indicated in Figure 6b. In total, we obtained 2876 PIFs for completely clear images, and by careful inspection, the PIFs selected by our method mainly comprised the artificial river levee of the Qiantang River, the old Hangzhou City, and its surrounding village. Obviously, the selected PIFs remained unchanged throughout the study period, especially the river levee, which remained stable without any significant variations. The thresholds for excluding the cloudy images are $CP_{\min}=100$ and $T(R^{2})=0.8$, and 190 scene images have been selected for further radiometric normalization. 

**Figure 7:**
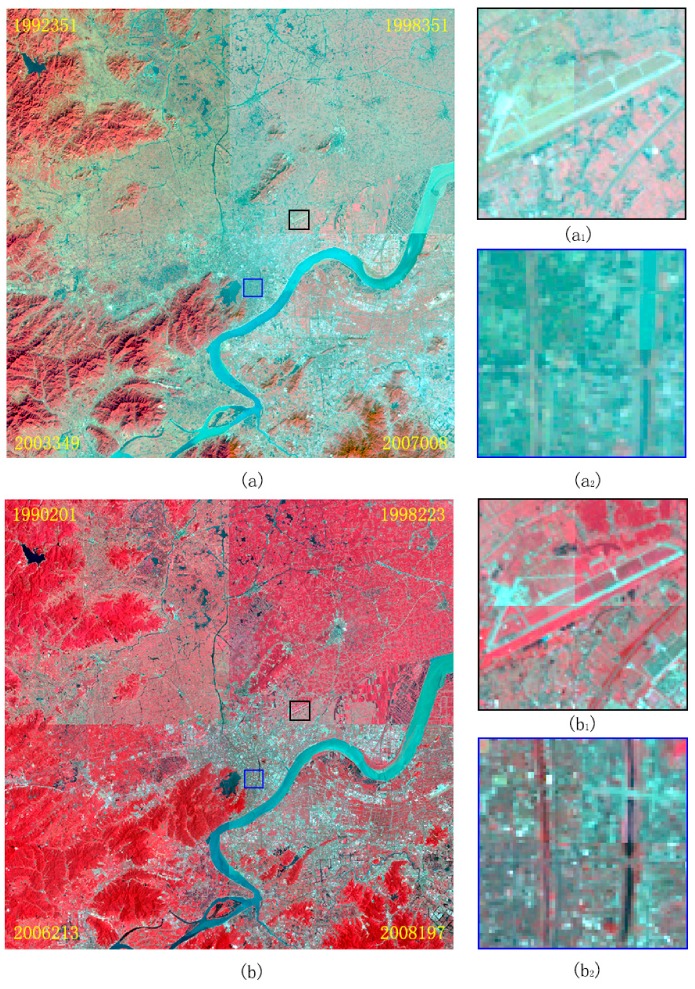
Resultant images of radiometric normalization. Panels (**a**,**b**) are the mosaicked results by four images corresponding to Figure 2. Panels (**a1**,**a2**,**b1**,**b2**) are two local areas that show very similar radiometric features, indicating the effectiveness of our method. Note that the color difference between vegetative land cover indicates that our method can maintain the radiometric signal, whereas no or little color difference between artificial objects (such as buildings and airports) indicates that our method can remove radiometric distortion.

The results of radiometric normalization corresponding to the images in Figure 2 are shown in Figure 7 with a mosaic pattern. In the original images of Figure 2, all the surface features, including buildings, roads, water bodies, vegetation, and wetland, exhibit an obvious radiometric difference. However, in the normalized image, the radiometric characteristics of land surface features such as buildings, roads (namely, PIFs), and water bodies are characterized with similar color and contrast in temporal-adjacent images, which indicates that the radiometric distortion has been eliminated effectively. The vegetation pixels acquired with similar acquisition dates in different years are characterized with distinct radiometric features, which demonstrates that the radiometric variations caused by seasonal changes of vegetation (important information contained in remote sensing images) have been effectively reserved. These results indicate that the method proposed in this paper is not only able to effectively eliminate the noise caused by random factors but also can maintain the time-dependent radiometric feature information of vegetation, thus providing good consistency of radiometric features for subsequent land use change detection and urban dynamic analysis.

**Figure 8:**
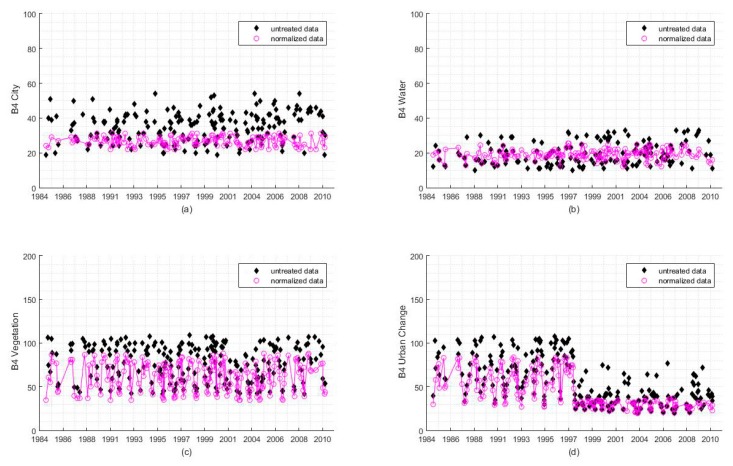
Comparison of the gray-scale values before and after radiometric normalization in long time-series sequences. Panels (**a**–**c**) present the comparison for three different land cover types: city, water body, and vegetation. The land cover did not change during the time period of observation. Panel (**d**) indicates radiometric normalization results for an area with changes in land surface. The pixel before 1997 corresponds to vegetation, whereas the pixel has changed to urban buildings after 1997.

Figure 8a–d show the scatter diagrams for the variations of gray-scale values from the near-infrared Band (Band 4) over time for four pixels with city, water body, vegetation, and “vegetation to city” land cover types. In this figure, the black points indicate the original gray-scale values, while the red points represent the gray-scale values after radiometric normalization. In Figure 8a, the original gray-scale values contained noise that led to large magnitude fluctuations, which increased the difficulty of discovering the variation pattern. The time-series’ gray-scale values after radiometric normalization lie close to a straight line, demonstrating that the radiometric distortion is effectively eliminated. The water body pixel in Figure 8b is similar to the city pixel in Figure 8a, although the magnitude of variation is smaller than that of the city pixel. Figure 8c shows the variation curve of the gray-scale value for the vegetation pixel over time, and the radiometric distortion at different times is effectively eliminated. The results of radiometric normalization can well describe the inter-annual variation of vegetation. Figure 8d shows the gray-scale value change of a pixel where the vegetation was converted to city in approximately 1997. The data transformation from vegetation has an obvious periodic variation with respect to a straight curve, and the results of radiometric normalization can enhance the points of discontinuity, thus providing the basis for accurately timing the land cover change. These results indicate that the radiometric normalization method proposed in this paper can enhance the time-series’ characteristics for various types of pixels and can provide comparable time-series data for further applications.

### 5.2. Evaluation and Comparison of the Experimental Results

Because the workload for evaluating all available pairwise PIF selections is huge, only part of the images are selected to perform an accuracy evaluation. First, to test the normalization effect of long time-series of images, 26 images were collected on approximately the 100th days of different years under smaller cloud effects for experiment 1. Second, to test the normalization effect of short time-series of images, 11 images are selected with a proportion of clear pixels greater than 50% during 2001 and 2002 to conduct experiment 2.

As the difference between our method and traditional methods lies in the PIF selection and the normalization strategy, we therefore evaluate our method from these two aspects.

#### 5.2.1. Evaluation of Pseudo-Invariant Features Selection

For the contrast method of PIF selection, the IRMAD PIF selection method has been widely used for radiometric normalization and achieved satisfactory results for a number of applications [9]. However, the IRMAD PIF selection method can only consider images obtained at two different dates for the same area for one time. We should classify the images to be processed into multi-groups for radiometric normalization to obtain PIFs. First, we manually selected an image with good radiometric quality as the reference image, and then the IRMAD method was used to determine the PIFs between the image to process and the reference image one by one. The confidence parameter for IRMAD is 95%. Obviously, only the PIFs selected by all groups can serve as the final PIFs for time-series image radiometric normalization, which can be expressed as follows:

(22) $$G_{S}=|G_{1}\cap G_{2}\cap ...\cap G_{g}|,$$

where $G_{i},i=1,2,..,g$ represents the PIFs selected by the image pair of group i; g indicates the group number of the image pair, which is one less than the image number; and $G_{s}$ is the PIFs selected by all groups. However, the number of pixels in $G_{s}$ is as small as 10 for both experiment 1 and experiment 2. The small number of obtained pixels indicates that the PIFs selected by the IRMAD method presents considerable uncertainty when used for long time-series PIF selections, especially with a large number of groups. Thus, sufficient PIFs cannot be obtained across all groups using the IRMAD method.

#### 5.2.2. Evaluation of Normalization Strategy

To contrast normalization strategy, we adopted two strategies. The first strategy was to select one reference image and then to normalize other images to the reference image one by one (the contrasting method 1). Rather than selecting one image from the time-series images, the second strategy was to use a synthetic image composited of the mean gray-scale value of the selected PIFs [34] (the contrasting method 2), which could avoid the difficulty of reference image selection. Then, according to the obtained PIFs using our method, we carried out radiometric normalization using either our method or the two contrasting methods and evaluated the overall residual of the radiometric normalization.

To evaluate the optimized strategy, we calculated the root mean squared error $\mathrm{RMSE}(X_{i},X_{j})$ between image $X_{i}$ and image $X_{j}$, which can be expressed as

(23) $$\mathrm{RMSE}(X_{i},X_{j})=\sqrt{\frac{1}{m}{\sum_{s\in S}\left|f(x_{i}^{s})-f(x_{j}^{s})\right|}^{2}},$$

where *S* represents the set of PIFs, m represents the number of PIFs, $f(x^{s})$ represents the gray-scale of pixel $x^{s}$ after correction. Smaller values correspond to a better correction effect for two scenes in the images and vice versa.

We also statistically analyzed the average and standard deviations of the RMSE to evaluate our method and its contrasting methods in general, and these parameters were calculated as follows:

(24) $$\mu (\mathrm{RMSE})=\frac{1}{n^{2}}\sum_{i=1}^{n}\sum_{j=1}^{n}\mathrm{RMSE}(X_{i},X_{j}),\;\mathrm{and}$$

(25) $$\sigma (\mathrm{RMSE})=\sqrt{\frac{1}{n^{2}}\sum_{i=1}^{n}\sum_{j=1}^{n}{\left(\mathrm{RMSE}(X_{i},X_{j})-\mu (\mathrm{RMSE})\right)}^{2}},$$

where n represents the image number in the time-series. Because the shared PIFs selected by the IRMAD method in both experiments were too few and the representativeness was insufficient, the experiment used the PIFs selected by our method.

It is worth noting that the parameters’ adjustment process in step 4.4 affected the calculated RMSE. If the stretch parameter of $1/k_{min}$ was used, the new RMSE (i.e., with parameter adjustment) was proportional to the original RMSE (i.e., without parameter adjustment) with proportionality coefficient of $1/k_{min}$. Additionally, by comparing different results from experiments 1 and 2, we found that it was equivalent to normalize other images to the first image and the previously corrected image with a stretch process, and the coefficient $1/k_{min}$ was close to 1. The error variation due to the parameter adjustment was small. Despite this fact, we set $k_{n}$ = 1 to avoid its negative effect on error variation induced by parameter adjustment.

**Figure 9:**
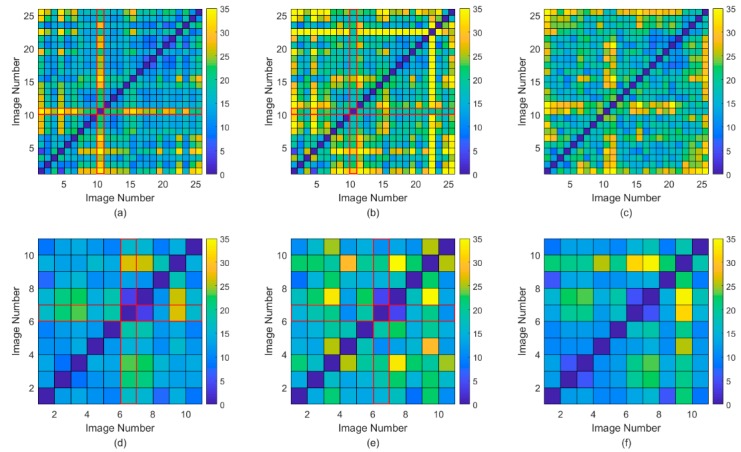
Error matrices of the Root Mean Squared Error (RMSE) using the proposed method and the two contrasting methods. As the color transitions from yellow to blue, the residual gradually decreases. (**a**) Error matrix of our method for experiment 1; (**b**,**c**) represent error matrices of the two contrasting methods for experiment 1; (**d**) error matrix of our method for experiment 2; (**e**,**f**) represent error matrices of the two contrasting methods for experiment 2.

The error matrices measured by the RMSE of our method and the contrasting methods are shown in Figure 9. In the figure, as the color changes from yellow to blue, the RMSE gradually decreases. This figure shows the following. (1) The error of contrasting method 1 on the row or column of the reference image (marked by a red edge) is obviously smaller, which occurs because the optimization objective of contrasting method 1 is to minimize the residual between the correction results of various scenes of the image and the results of the reference image, whereas in our method, the error is larger because our method begins with the maximum standard variation. (2) The error of the contracting methods between various scenes is larger than that of our method, and many yellow mosaics are observed. (3) The error distribution for our method is smoother, and the error distribution for the entire scene of the image is more uniform, indicating that our method has more homogeneous correction results.

**Figure 10:**
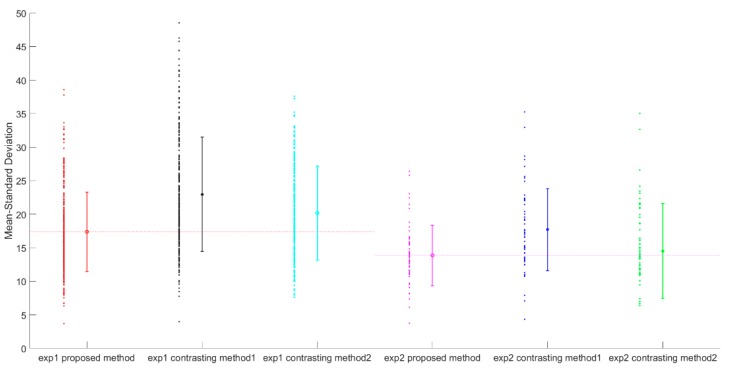
Means and standard deviations of the RMSE for our method (the optimized normalization strategy) and the two contrasting methods (the traditional normalization strategy with one reference image and the virtual reference image made up of the mean value); the left-side points indicate the RMSE between a single image pair, and the right-side lines indicate the means and standard deviations of the RMSE. Exp1 and exp2 represent experiment 1 and experiment 2, respectively.

Figure 10 shows the means and standard deviations of the RMSE of our method and the contrasting methods. The results show that the means of the RMSE of our method (17.39 and 13.87) in both experiment 1 and experiment 2 are smaller than those of contrasting method 1 (22.97 and 17.73) and contrasting method 2 (20.16 and 14.54), which indicates that the normalized gray-scales of our method are more tightly distributed. In addition, the standard deviations of our method (5.93 and 4.51) are smaller than those of contrasting method 1 (8.51 and 6.12) and contrasting method 2 (7.00 and 7.05) in experiment 1 and experiment 2, which indicates that the error distribution for our method is more uniform and the obtained time-series’ gray-scale curve is smoother. These features indicate that our method can overcome the steep rise and steep fall in the profiles of the gray-scale value.

#### 5.2.3. Comparison with the Reflectance

Because the time-series of radiometrically normalized images do not have the same physical meaning as those that we have regularly used, such as radiance or reflectance, how to interpret the radiometric normalized result remains a challenge. It’s a good choice to compare our result with the bottom-of-atmosphere (BOA) reflectance, which has removed atmospheric distortion. However, we lack the parameters required for carrying out atmospheric correction and obtaining the BOA reflectance. Thanks to the fact that the Landsat images are distributed with an improved metadata file, which includes parameters for transforming a digital number (i.e., gray-scale value) into top-of-atmosphere (TOA) reflectance [35]. Due to its easy availability, the parameters have been widely used for remote sensing applications, so we used the TOA reflectance as a baseline to evaluate our method and contrasting method 1.

Because the time-series gray-scale value and TOA reflectance have different physical units, it is difficult to use an absolute measure to qualify their difference. The correlation coefficient CC describes the linear correlation between two variables with different measures, which indicates the reliability that one can estimate one variable with the other variable using a linear equation. Therefore, we used a correlation coefficient to evaluate the performances of our method and contrasting method 1. Let $f(X_{O}^{l})=<f(x_{O1}^{l}),f(x_{O2}^{l}),...,f(x_{On}^{l})>$ and $f(X_{C}^{l})=<f(x_{C1}^{l}),f(x_{C2}^{l}),...,f(x_{Cn}^{l})>$ represent the normalized time-series gray-scale values of the *l*th pixel obtained by our method and contrasting method 1, respectively, $f(Y^{l})=<f(y_{1}^{l}),f(y_{2}^{l}),...,f(y_{n}^{l})>$ indicates the time-series TOA reflectance; then, we calculated the correlation coefficients $CC(f(X_{C}^{l}),f(Y^{l}))$ and $CC(f(X_{O}^{l}),f(Y^{l}))$, respectively.

**Figure 11:**
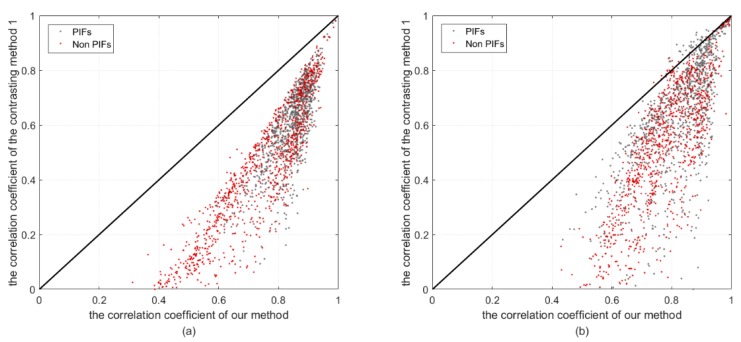
The correlation coefficient comparison of two experiments; (**a**,**b**) represent the results of exp1 and exp 2, respectively. The abscissa axis represents the correlation coefficient between the time-series Top of Atmosphere (TOA) reflectance and the normalized gray-scale value obtained by our method, and the longitudinal axis indicates the correlation coefficient between the time-series TOA reflectance and the normalized gray-scale value obtained by our contrasting method 1.

We randomly selected 1000 PIFs and 1000 non-PIFs (such as vegetative or land-cover changed pixels) to compute the correlation coefficient, and the results are shown in Figure 11. We found that the correlation coefficient $CC(f(X_{O}^{l}),f(Y^{l}))$ was larger than $CC(f(X_{C}^{l}),f(Y^{l}))$ in most cases; the means for exp1 and exp2 by our method reached 0.781 and 0.793, respectively, whereas the means for the contrasting method for exp1 and exp2 were 0.508 and 0.562, respectively. The statistical value demonstrated that the results of our method were more highly correlated with the TOA reflectance, which indicates that our result can be transformed to the TOA reflectance using a linear equation with a high confidence. Additionally, the results indicate that our method can provide a comparable result with the TOA reflectance, which provides an alternative method for radiometric correction under the situation that the absolute radiometric correction parameters cannot be obtained.

## 6. Discussion

Our method for the radiometric normalization of long time-series Landsat images has the following innovations.

The identification of inliers (namely, normal observation values with good acquisition conditions) and outliers (namely, abnormal observation values caused by noise such as clouds and cloud shadows) in time-series of observation values for various pixels forms the basis for the radiometric normalization and further applications. As the number of images used for long time-series is very large, cloud detection is a time- and labor-intensive process. We captured the distinctive feature that variations in the gray-scale values of a clear pixel are concentrated in a narrow range, whereas the variations in the gray-scale values of outliers are much larger than those of clear pixels. We introduced the IBCD method for outlier identification, which alleviates the time and labor costs and obtains an acceptable result. Similarly, we selected clear-sky PIFs according to a novel measurement, namely, the slope of the clear line segment in the sorted time-series profile, which can exclude the negative effect of noisy pixels. This method can consider all the observations in the time-series images instead of just a small number of pixels, which increases the robustness of our method for undetected noise.

One objective of the time-series analysis is to maintain consistency among the observation values obtained at different times while reducing rapid up-and-down fluctuations between adjacent observations. The traditional correction method reduces the residual between the reference image and the image to be corrected, without constraining the residual between other images to be corrected. Our method, which is a typical greedy algorithm, takes all the corrected images as a reference for the images to be corrected, thus minimizing the residual for correction after adding each new image. The results also indicate that the residual obtained by our method is smaller than that of the contrasting method, and the observation values of the time-series are also smoother, which indicates the effectiveness of our method.

Our method can automatically determine the sequence of radiometric correction, which is very important for radiometric correction. If the gray-scale value distribution of the reference image is concentrated, while the gray-scale value distribution of the image to be processed is scattered, the gray-scale resolution of the images to be processed will be compressed compared with that of the reference image, and radiation information may be lost. However, in this paper, we developed a method that sorts the standard deviations of the gray-scale values of the PIFs in descending order. This approach guarantees that images with a wide gray-scale distribution are always corrected earlier and images with a narrow gray-scale distribution are corrected later. The image corrected later is stretched relative to that corrected earlier. The results of this strategy also indicate that the correction slope obtained by our method is generally greater than 1 (i.e., is relatively stretched). Finally, for the possible situation with a slope smaller than 1, we adjusted the parameters to ensure that the radiation resolution maintained a relatively large gray scale without compressing any pixels.

Additionally, some uncertainty may induce a decrease in the time-series normalization performance. Clouds and cloud shadows are important sources of noise in passive remote sensing and identifying abnormal observation values from time-series observation data is the basis of various applications. In this paper, according to the large fluctuation range of the gray-scale value caused by clouds, we developed a method based on the IBCD method to automatically identify the inliers and outliers to suppress the negative effect of contaminated pixels. Water bodies exhibit low reflectance in the near-infrared band and separating them from cloud shadows may be difficult; similarly, dense vegetation exhibits a high reflectance in the near-infrared band, and separating such vegetation from clouds may be difficult. For the PIFs (such as bare land, buildings, and roads), the gray-scale values in the near-infrared band are between those of low-reflectance water bodies and high-reflectance vegetation. Our method can easily differentiate abnormal observation values when the land cover type is PIFs. Therefore, the influence of these two deficiencies on the selection of PIFs is small.

From the image of the slope of the clear line segment, we can extract the PIFs using the threshold segmentation. At present, identifying PIFs requires manual trial-and-error to adjust the threshold of the slope, and they are evaluated and adjusted according to the obtained result. The unappropriated threshold setting may lead to failure in PIF selection. The time interval of the time-series of remote sensing images is long, and urban sprawl is dramatic in the research area. In a strict sense, no pixel is free from any changes. A PIF is a variable that cannot be precisely defined but instead refers to a type of pixel with a small variation magnitude.

Additionally, due to clouds, cloud shadows, and other noise factors, images have different numbers of clear PIFs, which may induce uncertainty in radiometric normalization. In the worst case, an image may not have sufficient PIFs to estimate a normalization equation, which will cause the image to not be processed. However, images that cannot be processed have large proportions of clouds or fewer clear pixels, and abandonment of these images will not result in a loss of much information.

Additionally, our method has certain drawbacks that may impede its application as follows: (1) this method needs relatively invariant pixels in the research area and thus has poor application potential in research areas that are completely covered by forests and farmlands; (2) in a region of rapid urbanization, the PIFs also experience variations in various radiometric characteristics, and many uncertainties are observed when there are changes in the entire time-series; (3) a long time-series is required to present the gray-scale curve of the images, which is unsuitable for short time-series; and (4) when one or more images have been accurately calibrated to reflectance, one intuitive method is to normalize other images to the corrected images, which provides the explicit physical meaning to the radiometric normalized result. However, our method cannot directly normalize the other images to the corrected image.

## 7. Conclusions

In this paper, we have proposed a radiometric normalization method for long time-series of remote sensing images, which exhibits favorable merits such as automatic outlier exclusion, PIF selection, and a novel strategy to minimize the RMSE between the image to be processed and the previously corrected images. In addition, we tested the method using long time-series of remote sensing data acquired by Landsat 5 TM for Hangzhou City. For experiments 1 and 2, the mean RMSEs of the images in the time series dropped from 22.97 and 17.73 (by contrasting method 1) to 17.39 and 13.87 (by our method), respectively, the standard deviations dropped from 8.51 and 6.12 to 5.93 and 4.51, respectively, and the means of the correlation coefficients between the time series gray-scale values increased from 0.508 and 0.562 (by contrasting method 1) to 0.781 and 0.793 (by our method), respectively, reflecting a significant performance gain by our method.

Additionally, the result indicates that our method can effectively eliminate differences in radiometric features between images and improve comparability between images. Moreover, the biophysical information from image time-series is well preserved, showing a smooth gray-scale value curve after radiometric normalization. The comparison between our method and the radiometric calibrated image demonstrates that our method provides a promising alternative method for radiometric normalization, especially when the parameters needed for absolute radiometric corrections are absent.
